# The effects of the DHACA method on expressive communication in children with autism spectrum disorder

**DOI:** 10.1590/2317-1782/e20240148en

**Published:** 2025-04-07

**Authors:** Fernanda Cristina de Oliveira Luna Barbosa, Ana Cristina de Albuquerque Montenegro, Bianca Arruda Manchester de Queiroga

**Affiliations:** 1 Programa de Pós-graduação em Saúde da Comunicação Humana – PPGSCH, Universidade Federal de Pernambuco – UFPE - Recife (PE), Brasil.

**Keywords:** Autism, Communication, Speech, Language and Hearing Sciences, Assistive Technology, Alternative and Augmentative Communication Systems

## Abstract

**Purpose:**

This study aimed to assess the contributions of the DHACA method to expressive communication development in children with autism spectrum disorder (ASD).

**Methods:**

This longitudinal case series study had a sample of 12 children with ASD, nonverbal or minimally verbal communication, and support level one or two. Data were collected by applying the ACOTEA-R Protocol by analyzing videos recorded during intervention sessions before and after using the DHACA. Participants underwent 20 individual speech-language-hearing sessions with the DHACA.

**Results:**

After the intervention with the ACOTEA-R, 10 of the 12 children improved their overall expressive communication skills. Concerning the communicative profile, initially, 10 children were nonverbal and 2 were minimally verbal. After the intervention, 7 evolved to a verbal pattern, whereas 5 remained nonverbal. The progress of the following communication skills stands out: use of sentences with four or more words, naming objects, social expressions, greeting people, and making comments. Moreover, 8 of the 12 participants advanced to the third skill in the DHACA, characterized by request with lexical and morphosyntactic expansion.

**Conclusion:**

The children’s speech and use of the communication book indicated progress in their expressive communication development after intervention with the DHACA.

## INTRODUCTION

Autism spectrum disorder (ASD) is a neurodevelopmental disorder that affects one in 36 children worldwide, characterized by persistent impairment in reciprocal social communication and interaction, and restricted and repetitive patterns of behavior, interests or activities^(^1^)^.

Communication impairment is one of the characteristics necessary for its diagnosis, possibly accompanied by developmental delays and completely absent oral language. Studies highlight communication deficits as important and characteristic ASD impairments^(^2^)^. The greatest language difficulties faced by children with ASD are associated with pragmatic aspects and narrative structure – hence, social communication difficulty is a central impairment in ASD^(^3^)^.

It is estimated that approximately 30% of children with ASD remain with nonverbal or minimally verbal communication even after years of intervention. Although the precise definition of “minimally verbal” is still limited, recent studies have conceptualized it as greatly restricted expressive communication, predominantly using isolated words or gestures^(^4^)^.

Thus, there is a need to provide these children with an alternative communication resource that enables the initiation, maintenance, and expansion of dialogical interactions. Such a resource must consider their specific clinical inabilities with shared attention, gaze direction, and intentionality^(^5^)^. This approach is crucial, as it contributes significantly to filling communication gaps and fostering more active and inclusive social participation.

Augmentative and alternative communication (AAC) is a form of assistive technology (AT) that provides greater autonomy and independence. This area of ​​knowledge offers various techniques, resources, and strategies to temporarily or permanently compensate for difficulties in individuals with severe disorders and help them understand and use communication^(^6^)^.

This study used the DHACA method (Development of Communication Skills in Autism), an AAC instrument aimed at stimulating and developing functional communication and aiding speech-language-hearing therapy for children with ASD without functional speech. Its theoretical premise is the Sociopragmatic Theory, which emphasizes sociopragmatic aspects in acquiring and developing linguistic skills^(^7-11^)^.

The DHACA is a pioneer method in Brazil due to its original use of a robust AAC system to develop functional communication in individuals with ASD – a gap in national research. The implementation of this method aims to provide a more effective means of communication, contributing significantly to the learning, social inclusion, and improvement of the quality of life of nonverbal individuals with ASD and their families.

The flexibility inherent to DHACA, allowing adaptations to each child’s needs, is also an important differentiator, considering the diversity of ASD cases. Unlike many foreign methods used in the country, DHACA offers a culturally relevant and accessible option aligned with Brazilian reality, increasing the possibility of successful interventions^(^8-10^)^.

**Figure 1:**
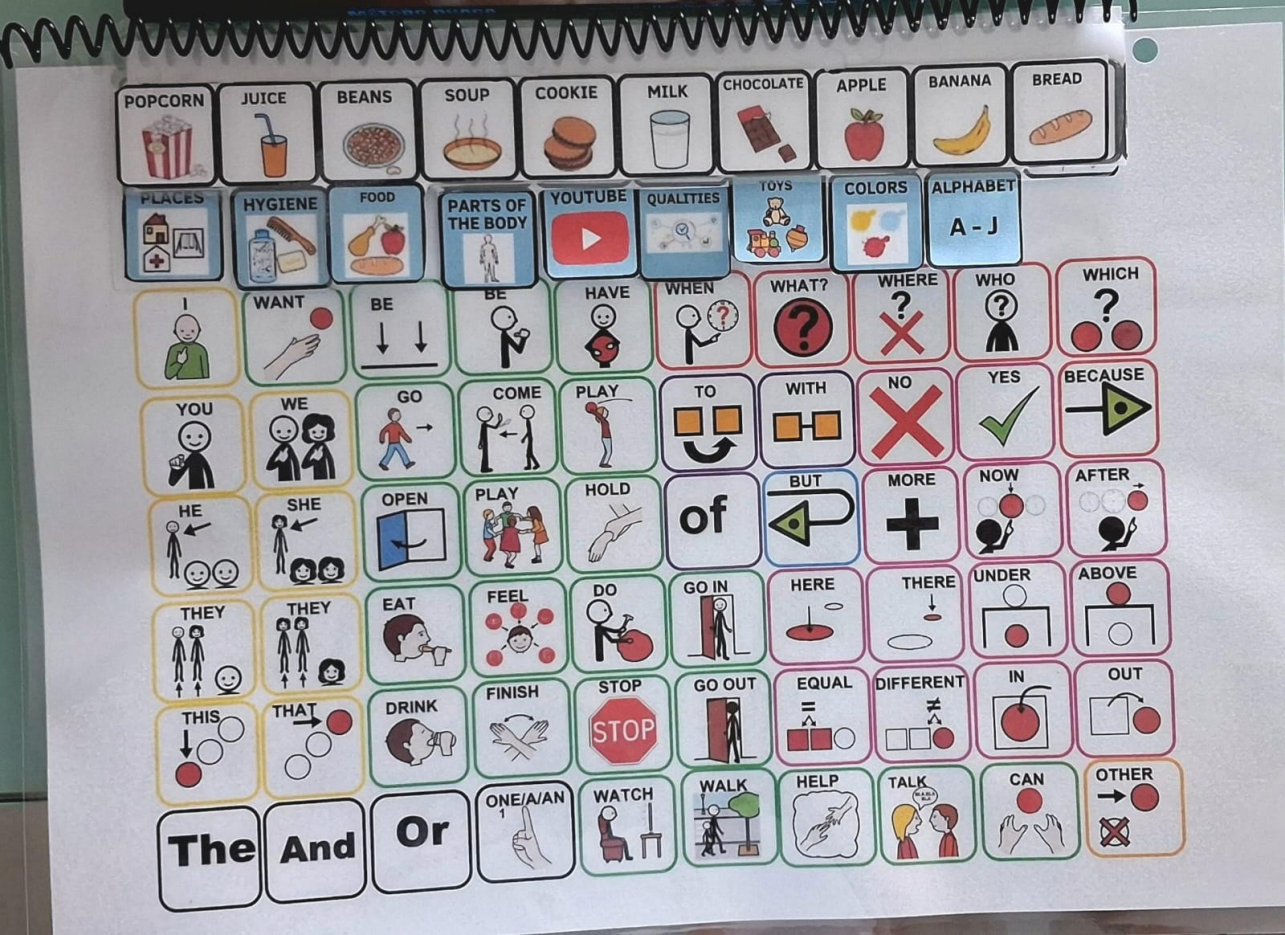
. DHACA book

**Source**: The author (2023)

The DHACA assumes that linguistic signals are acquired through daily interactions between subjects, and their development is guaranteed by shared attention and the gradual understanding that interlocutors are intentional agents. DHACA details the communication skills to be developed through the book with a robust AAC system^(^11^)^ (Figure 1).

This study aimed to evaluate the effects of the DHACA method on expressive communication development in children with ASD.

## METHOD

This is a quantitative longitudinal case series study, following the evolution of data throughout the investigation period.

This study is integrated into the research project of the Federal University of Pernambuco (UFPE) entitled, “SPEECH-LANGUAGE-HEARING THERAPY AND AUTISM – KNOWLEDGE, INTERVENTION, AND INCLUSION”, registered and analyzed by UFPE’s Ethics Committee, following resolution no. 466/12 of the National Health Council, and approved under protocol no. 2.106.800.

This research and all its procedures were conducted at a speech-language-hearing teaching clinic from March to October 2022. The study sample comprised 12 children with ASD, aged 3 to 6 years, of which 2 were females (17%) and 10 were males (83%).

The inclusion criteria were being 3 to 6 years old (given the relevance of intervening during early childhood language development) and diagnosed with ASD (according to the criteria outlined by the American Psychiatric Association in the 2015 DSM-V)^(^12^)^.

The exclusion criteria were children with ASD with verbal communication or with comorbidities (e.g., malformations, associated genetic syndromes, neurological changes, and physical, intellectual, auditory, and visual disabilities).

All participants’ parents/guardians were informed about the study’s methodological procedures, voluntarily signed an informed consent form, and answered a medical history survey and the Preference Item Assessment (PIA)^(^13^)^.

Participants were evaluated with the Communication Assessment in Children with Autism Spectrum Disorder (ACOTEA-R)^(^14^)^.

The intervention had 20 weekly 45-minute individual speech-language-hearing sessions, meticulously planned and structured by the therapists. They considered each child’s preferences, based on information collected with the PIA before beginning the interventions, to personalize their therapy according to each participant’s specific characteristics.

The sessions were conducted by undergraduate and postgraduate speech-language-hearing students at UFPE, trained in using the DHACA method, and supervised by the professionals who designed the method (they observed the intervention through a “spy mirror”). They used semi-structured playful activities to set an environment conducive to developing communication skills, encouraging participants to engage actively in communicative interactions^(^11^)^. They also used the DHACA communication book with 66 essential vocabulary pictograms on a single page and smaller overlapping pages with a line of 10 accessory vocabulary pictograms, arranged on separate pages according to the lexical category, gradually inserted during the therapeutic process.

During the activities, the items of interest to the participants were positioned within their visual field, and they were encouraged to express themselves by pointing to the relevant pictograms in the communication book. They initially used a full physical cue to teach the child to point to the pictograms representing the initial sentence, gradually replacing it with partial physical, visual, and verbal cues until the child could perform the task independently. They also used the modeling strategy, in which the interlocutor demonstrates the construction of the desired sentence, providing a model until participants can express themselves autonomously, without needing any assistance. Moreover, they talked to the child using several sentences with the DHACA communication book.

Tangible guidance (e.g., full physical help or cues, partial physical cues, and gestural cues) optimizes the effective teaching of new skills to children diagnosed with ASD. This approach helps them understand more comprehensively and carry out the proposed activities, considering these children’s propensity to interact effectively with visual stimuli. Visual cues aim to reduce errors and increase access to preferred items. The cues were gradually removed according to each participant’s performance^(^11,15^)^.

Furthermore, the researchers met with the parents before starting the intervention to explain about ASD and AAC, answer questions, and hold a practical workshop on the DHACA method, using the DHACA book, modeling, and physical, visual, and verbal cues. The therapists also talked to the parents in all weekly sessions to get feedback on their children's development at home and school and guide them on modeling, the stimulated skills, and their generalized use in different communication situations. They often do activities with the parents during the session to get their feedback.

The DHACA describes communication skills and their specific goals in detail, as outlined below. Participants were encouraged to develop and acquire them sequentially^(^11^)^.

Initial communicative intention – The child must be able to present communicative intention by asking the interlocutor for something, pointing to the pictograms I + WANT (in the communication book) + a separate pictogram*.

Request with lexical expansion in accessory vocabulary – The child must be able to request something from the interlocutor, pointing to the pictograms I + WANT + a pictogram from the optional vocabulary in one of the two tabs of specific lexical categories.

Request with lexical and morphosyntactic expansion – The child forms sentences with the pictograms: I + WANT + two pictograms (from either the active or essential vocabulary).

Morphosyntactic, lexical, and communicative function expansion – The child forms sentences with three or more words, with different objectives: Development of communicative functions: 1. Informing, asking – using questions with interrogative pronouns (who, when, which, where, etc.); 2. Commenting – making comments, giving spontaneous information, demonstrating something, showing pain, giving an opinion, expressing ideas; 3. Expressing feelings and gratitude; 4. Social interaction – greetings, farewells, thanks, apologizing, and showing off. Sentences are constructed sequentially by pointing to the pictograms and may be accompanied by speech. 5. Dialog – the child presents the following communicative functions: Reporting – telling a fact or retelling stories; Imagination – creating a story or telling jokes; Conversating – sustaining a conversation.

Research participants were reassessed after the intervention, collecting data with the ACOTEA-R^(^14^)^.

The researchers recorded each participant’s first three and last three sessions for data collection. Each video lasted an average of 45 minutes. This analysis used the ACOTEA-R to examine communicative behaviors^(^14^)^.

The lead researcher systematized the data, encompassing the medical history survey, protocols, evolution sheets generated throughout the speech-language-hearing sessions, and the video recordings of the evaluations made during therapy to fill out the ACOTEA-R^(^14^)^. The data were recorded and systematized in a Microsoft Excel spreadsheet. The next stage included a descriptive statistical analysis of the frequency of responses to interpret the results better. Each communication skill evaluated with the ACOTEA-R^(^14^)^ was coded with the letter P followed by a number (e.g., P1, P2, P3).

## RESULTS

This section presents the data analysis regarding ACOTEA-R scores of expressive communicative skills^(^14^)^ applied before and after the intervention with the DHACA.

**Figure 2:**
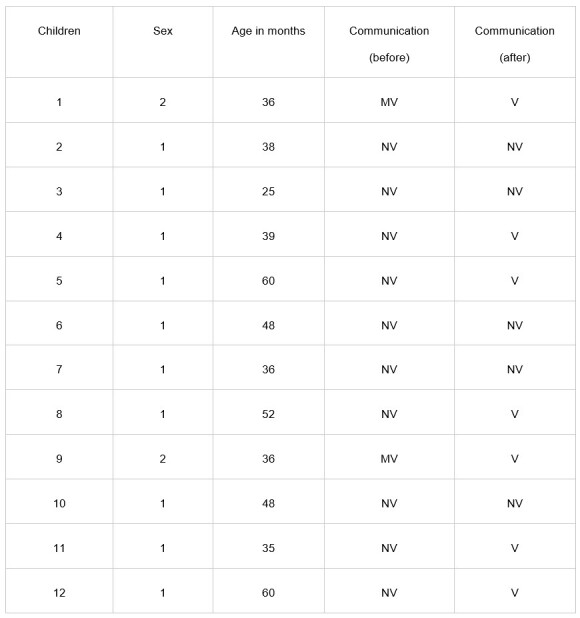
Result of the communicative profile before and after the intervention with the DHACA method. Recife, 2024

**Figure 3:**
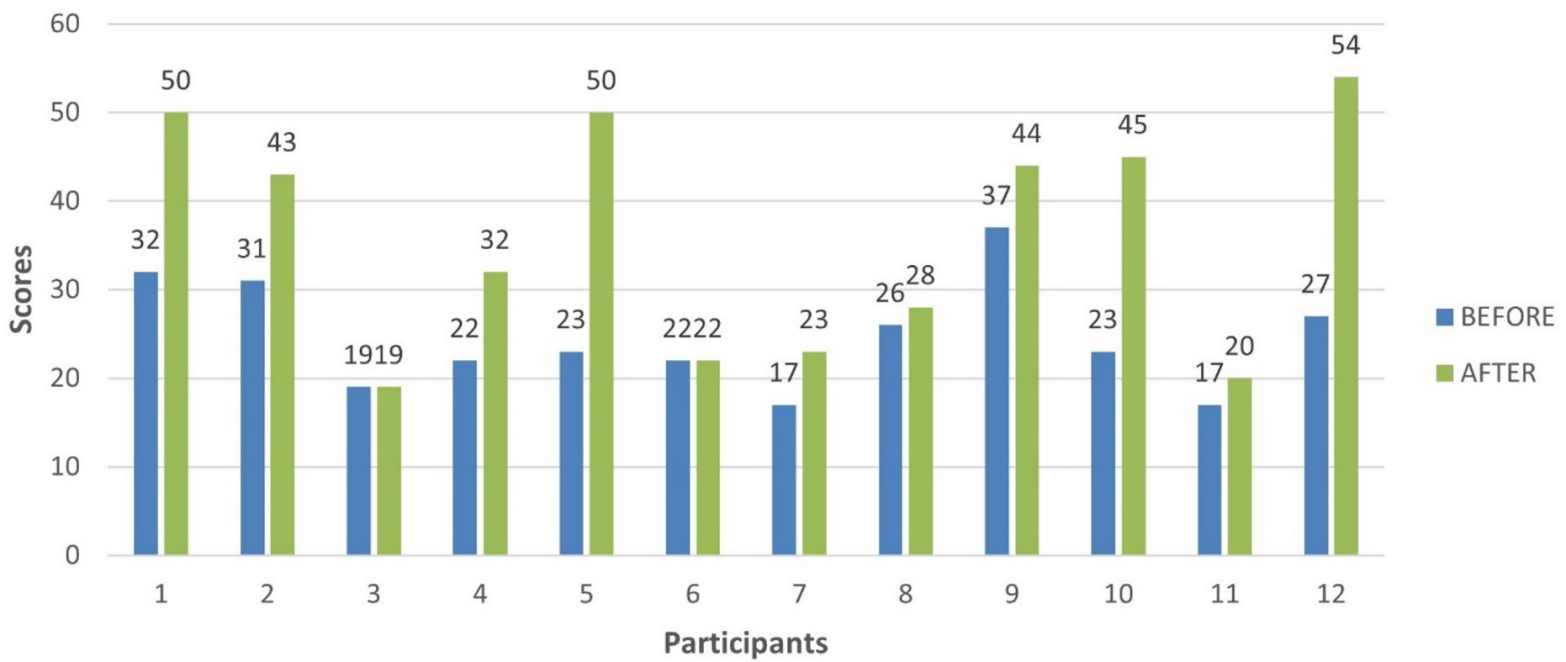
Scores of the expressive communication skills of children with autism spectrum disorder before and after the intervention with the DHACA method

**Source**: The author (2023)

Figure 2 shows the development of speech production. Figure 3 shows an increase in ACOTEA-R scores^(^14^)^ in 10 of the 12 participants. This means that the intervention with the DHACA helped most participants develop their expressive communication skills.

Nonverbal (NV); Minimally verbal (MV); Verbal (V); Sex – Male (1); Female (2)

Caption of the expressive communication skills assessed by ACOTEA-R: P1 = The child expresses discomfort; P2 = The child protests to show he/she does not want something; P3 = The child demonstrates he/she likes things; P4 = The child shows interest in other people; P5 = The child requests that an activity, gesture, or game continue when it is interrupted; P6 = The child asks when some object is taken from him/her or when he/she wants more food; P7 = The child calls the other person’s attention; P8 = The child asks for something that you have never offered him/her, but that is within his/her visual, auditory, tactile reach; P9 = The child requests objects that are not in sight; P10 = The child greets people; P11 = The child offers or shares things; P12 = The child shows something to you; P13 = The child asks for help; P14 = The child uses social productions (hi, thanks, bye); P15 = The child asks questions; P16 = The child names objects or people spontaneously or in response to your question; P17 = The child makes comments; P18 = The child uses sentences with four or more words; P19 = The child respects turns and maintains a conversation; P20 = The child does NOT have tantrums; P21 = The child smiles socially

Although two participants did not significantly increase their ACOTEA-R scores^(^14^)^, they improved specific skills. One of the participants progressed in five skills previously manifested through motor and verbal stereotypies, which were no longer evident in these same situations, and reduced the practice of pushing objects to request something from the other participant. Although these participants did not significantly improve their expressive communicative skills in the ACOTEA-R^(^14^)^, they gained in expressive communication.

**Figure 4:**
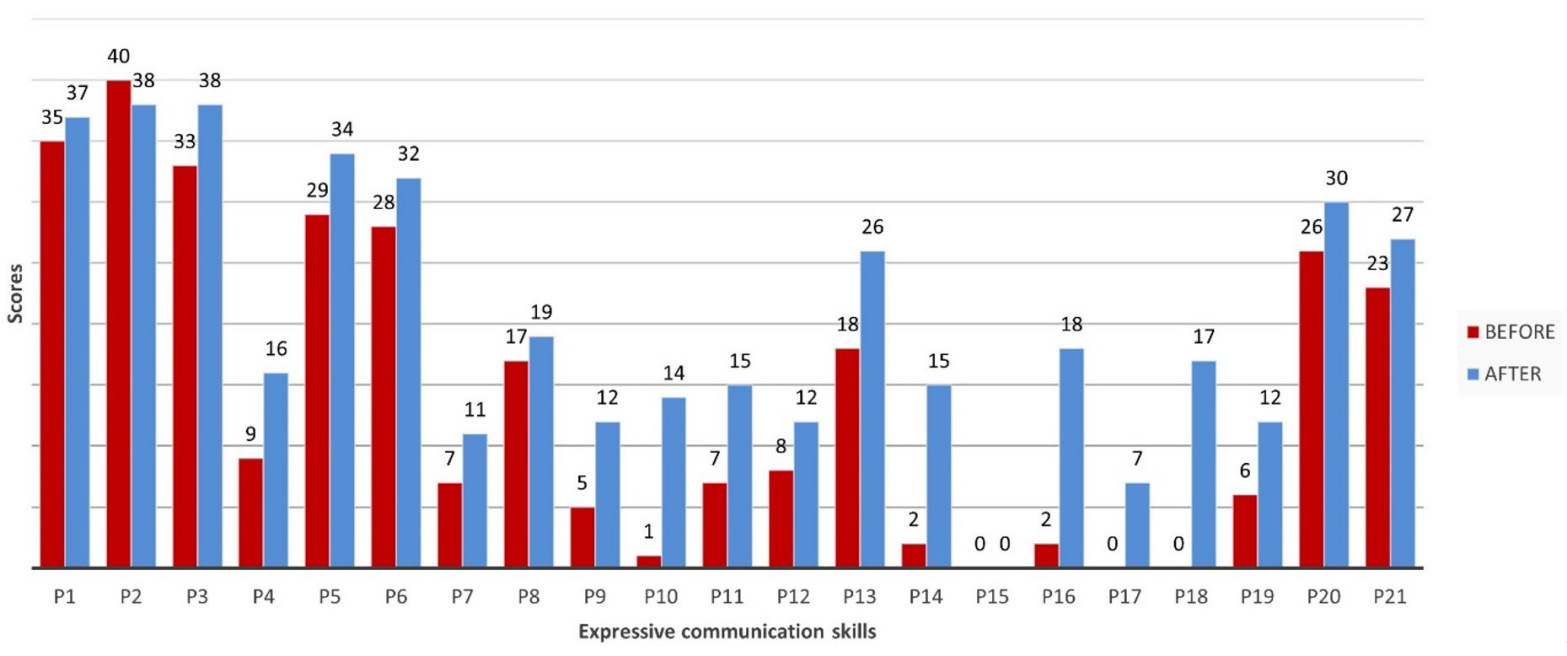
Total scores of the expressive communication skills before and after the intervention with the DHACA method

**Source**: The author (2023)

The analysis of the ACOTEA-R data^(^14^)^ on each expressive communication skill (Figure 4) highlights the increased frequency in the following communication skills:

P18 – Using sentences with four or more words: from 0 to 17.
P16 – Naming objects or people: from 2 to 18.
P10 – Greeting people: from 1 to 14.
P17 – Making comments: from 0 to 7.

Figure 3 shows an evolution in response forms. The “Speech” category was a notable indicator of progress, surpassing an initial frequency of 3 to a final 28. Other responses likewise had an increased frequency – e.g., “Pointing to a picture using the book without speaking” (from 0 to 33), “Pointing to a picture using the book with speaking” (from 3 to 28), “Gestures” (from 16 to 36), and “Handing over something” (from 20 to 30) (Figure 3).

**Figure 5:**
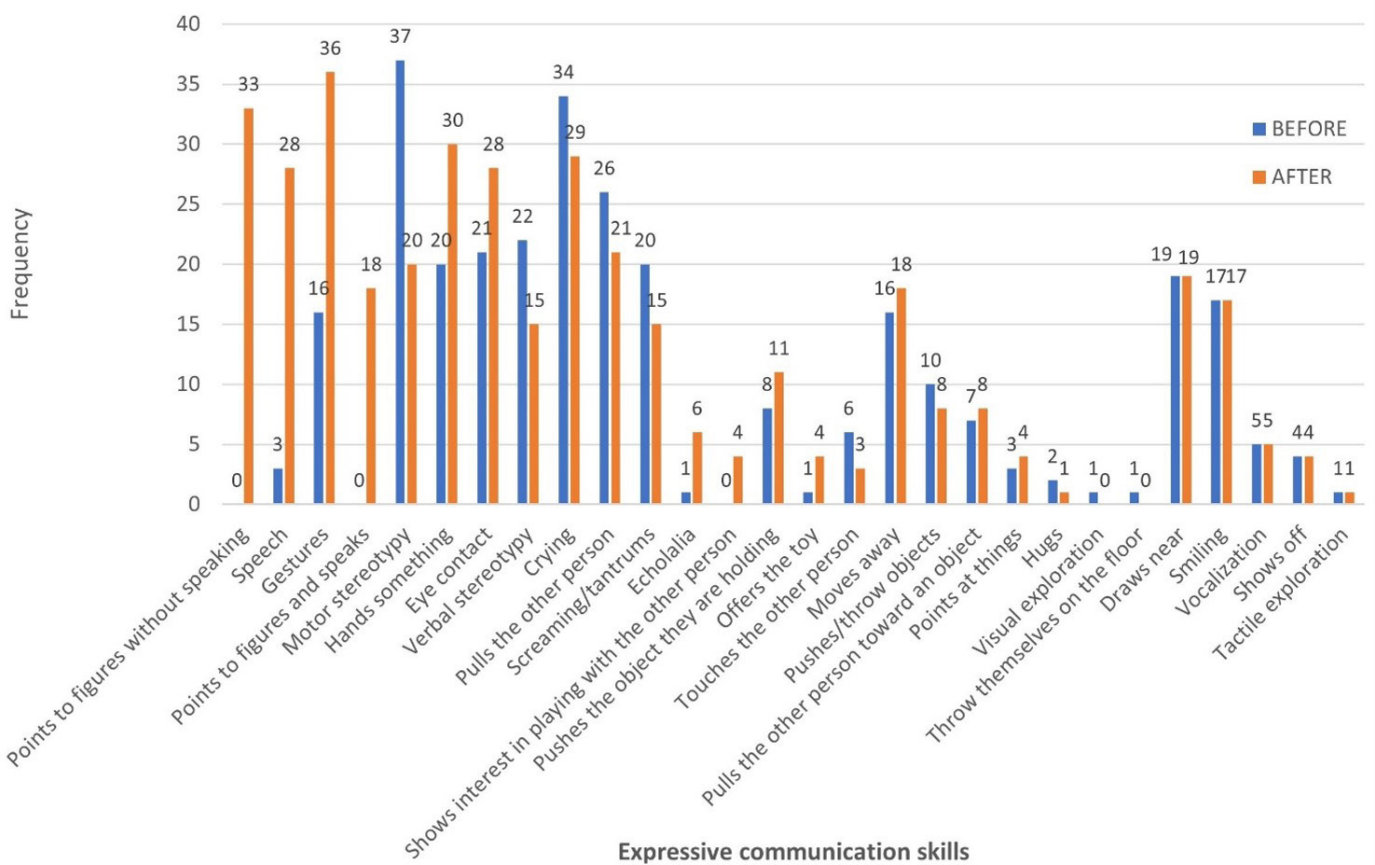
Expression modes for ACOTEA-R expressive communicative skills before and after the intervention. Recife, 2024

**Source**: The author (2023)

On the other hand, the rates of responses considered less functional decreased significantly (Figure 5). Some behaviors decreased in frequency, such as “Motor stereotypies” (from 37 to 20), “Verbal stereotypies” (from 22 to 15), “Pulling the other person” (from 26 to 21), and “Screaming/tantrums” (from 20 to 15), highlighting the effectiveness of the intervention in minimizing less adaptive behaviors.

The progress in developing expressive communication skills stands out among the aspects analyzed after the speech-language-hearing intervention. This was evidenced by an increase and emergence of speech, communication through AAC – pointing to figures (pictograms), using the DHACA communication book with and without speech –, using gestures, handing objects to express the desire to achieve or obtain something, and eye contact, as shown in Figure 5.

Figure 5 also shows the progress in producing functional speech spontaneously and through AAC, using figures (pictograms) through the DHACA communication book.

The ACOTEA-R^(^14^)^ results showed considerable progress in the communicative profile, as shown in Figure 2. In the initial assessment, only two children (16.6%) had a minimally verbal communication pattern, while 10 children (83.3%) had a nonverbal profile. However, the ACOTEA-R^(^14^)^ reassessment results after the 20 intervention sessions revealed that five children (41.6%) maintained a nonverbal communicative profile, while seven children (58.3%) transitioned to a verbal communication profile.

The development of communicative skills established by the DHACA progressed significantly. After the intervention, a remarkable 66.67% of participants advanced to the third skill of the method (characterized by request with lexical and morphosyntactic expansion), two (16.67%) participants achieved the penultimate skill (involving morphosyntactic, lexical, and communicative functions expansion), and another 16.67% of the children developed up to the second skill (request with lexical expansion in accessory vocabulary).

## DISCUSSION

The study results reveal improved expressive communication in children with ASD after intervention with the DHACA^(^11^)^, demonstrated by the increase in the overall ACOTEA-R^(^14^)^ score and the identification of expressive communication skills most frequently used by study participants. These findings provide insight into these children’s progress, highlighting the positive impacts and improvements in their expressive communication skills.

These scores increased to 83.33% of the participants. This shows that the intervention with the DHACA^(^11^)^ using AAC tools developed expressive communication skills in this study population, corroborating the international literature^(^16^)^.

Similarly, a recent case study investigated the potential of assistive technology to help improve communication skills in individuals facing such difficulties, resulting in significant progress in the participant’s communication skills. The study also highlighted a marked improvement in social interactions, using cordial forms of communication, and a decrease in the severity of the child’s ASD symptoms, from moderate to mild within the autistic spectrum^(^17^)^.

Communication and social interaction are described as the most impaired skills in children with ASD^(^18^)^. The data in Figure 4 in the present study reveal an evolution in communicative functions, resulting in more functional communication, as they could make comments, show things to others, greet people, and use social productions (e.g., hi, bye).

Regarding grammatical aspects, two children achieved the second skill of the DHACA (“request with lexical expansion in accessory vocabulary” – I + WANT + pictogram), 8 children achieved “request with lexical and morphosyntactic expansion” (I + WANT + two words), and two children achieved the fourth skill (“morphosyntactic, lexical and communicative function expansion”), with sentences with four or more words and several communicative functions. Figure 4 shows a significant increase especially in skill P18 of ACOTEA (“use of sentences with four or more words”) in 50% of the children, confirming a great development, as no child demonstrated this skill before the intervention.

A study whose scope was to investigate syntactic, morphologic, and vocabulary delays in ASD found that children had impaired syntactic and morphological skills^(^19^)^. Thus, the ability to use sentences with four or more words functionally – a skill outlined in the development of the DHACA – indicates that this method develops grammatical competence effectively.

It has been stated that the use of longer sentences can be understood as the acquisition of a more complex syntax, highlighting that this linguistic competence is related to social communication, affecting reciprocal social interaction^(^20^)^.

These results indicate improved expressive communication, evidencing greater linguistic robustness after implementing the DHACA method. This phenomenon corroborates the conclusions of a review that argues that communicative development is a positive result of intervention with AAC^(^16^)^

Furthermore, such intervention not only affects nonverbal communication but also boosts the development of oral production of words and sentences. In the present study, five children developed functional speech with or without the support of the DHACA communication book.

As demonstrated, the communicative skills of the DHACA method^(^11^)^ – which were acquired or progressed to higher levels after the intervention – include the ability to request for activities or games to continue, request objects when removed, express the desire for more food, ask for help, greet people, employ social productions, name objects spontaneously or in response to questions, and use sentences with four or more words.

Despite being among the first pragmatic functions, the skills of “requesting” (requesting for activities or games to continue, requesting objects when removed, expressing the desire for more food, asking for help) appear significantly often with the use of speech, AAC, or both, demonstrating the evolution of language used as a linguistic system.

The ability to name objects, people, and attributes spontaneously occurred more frequently. The data demonstrated significant growth, going from only one child naming objects before the intervention to seven children who acquired this ability after implementing the DHACA method^(^11^)^.

The ability to name objects implies understanding the other person's command, revealing the child's ability to respond appropriately to the request to name something. This process demonstrates the ability to name objects and understand the other person's communicative intention within a context. In addition, the skills of greeting people and using social expressions are significantly present, reflecting a dynamic social interaction in which both the child and the adult/therapist understand each other’s behavioral intention (intentional action) and intentional state (communicative interests).

AAC is implemented through interaction with others^(^11^)^. The children in this study did not use to communicate through either AAC or speech. However, expressive communication developed after intervention with the DHACA^(^11^)^, with mixed communication emerging, characterized by the simultaneous use of communicative categories (sentences produced by combining speech with the communication book simultaneously)^(^21^)^.

The results in expressive communication skills, shown in Figure 2, demonstrate the development of speech production. This corroborates data evidenced in a meta-analytic study, whose scope consisted of evaluating the impact of AAC on the speech production of individuals with developmental disabilities. This survey encompassed 23 studies, involving 67 subjects, and consistently found that the vast majority (89%) significantly advanced in speech production after implementing AAC^(^22^)^.

Functional speech development progressed, as its response frequency increased by 28 after the intervention. This ability was not demonstrated before the intervention, corroborating the authors’ findings^(^22^)^, whose analyses presented data suggesting that interventions using AAC can also have positive effects on natural speech production.

The comprehensive data analysis of functional communication development (Figure 5) highlights the reduction of more primary communicative behaviors, such as screaming, crying, and tantrums. This finding corroborates a recent study investigating the perspective of special education teachers regarding barriers and facilitators in AAC use with students with multiple disabilities^(^22^)^. The author highlights the presence of challenging behaviors often associated with communication difficulties. Individuals who face obstacles in expressing their needs commonly resort to these behaviors as a form of communication, which in turn hinders social relationships.

This study corroborates study^(^23^)^ that demonstrate the effectiveness of alternative communication as an assistive technology with the potential to stimulate speech development, facilitate the acquisition of language skills, and contribute to the learning process. Furthermore, the study highlights the significant impacts of AAC in promoting social inclusion.

Previous studies have highlighted the use of alternative communication tools to improve the communication skills of children with ASD and stimulate spontaneous communication^(^24^)^. The data from this research reinforce the claim that they demonstrate advances in functional speech production, both autonomously and with the support of the DHACA communication book, as shown in Figure 5.

Thus, it can be inferred that the DHACA method^(^11^)^ is an innovative Brazilian therapeutic approach to developing expressive communication, especially morphosyntax, in children with ASD.

Moreover, a review study aimed to synthesize contemporary evidence on AAC effectiveness. The results highlight important improvements in speech and pragmatics when using the alternative communication system. It also found that children who received intervention through AAC had significantly greater gains in functional vocabulary than their peers who were not subjected to this method^(^25^)^.

The DHACA method^(^11^)^ points out the importance of continuing activities at home, having family engagement in established guidelines, and using the DHACA book in the dialogical interaction between the child and communication partners in daily life, as these aspects influence the results. In this regard, two study participants did not progress significantly. However, their parents and caregivers reported not using the DHACA book continuously at home (recorded weekly in the progress sheets, immediately after the session), which may have negatively impacted these children’s progress, since regular and consistent involvement is crucial for the consolidation of skills acquired during intervention sessions. These two children’s lack of significant progress highlights the need for a collaborative approach when implementing AAC intervention programs, emphasizing the importance of active family participation and generalization of the skills learned in the child's daily life.

This finding reflects those of another recent study, which emphasized that the success of interventions in children with ASD is closely related to the family’s support and active participation in the process. Parental involvement not only facilitates the learning and application of an alternative communication system but also promotes greater generalization of communication skills to different contexts and everyday situations^(^26^)^.

The sample size may imply a limited representation of the diversity of responses, restricting the generalization of the findings. Therefore, a more comprehensive and conclusive understanding of the impacts of the DHACA method on the expressive communication of children with ASD requires further studies with a larger and more heterogeneous sample.

## CONCLUSION

The research results show how implementing the DHACA method significantly helped advance expressive communication skills in children with ASD, corroborating the initial hypothesis that guided this study. The results indicate a positive effect of the DHACA on expressive communication development in children with ASD, demonstrated in speech acquisition, morphosyntactic development, communicative intention, and other pragmatic functions. These results not only confirm the effectiveness of the DHACA method but also indicate its positive impact.

However, it is important to recognize the limitations inherent to this research. Its findings do not represent a definitive conclusion, but rather a starting point for future, more in-depth investigations. Despite the positive results, the intervention duration suggests the need for longer investigations for a more comprehensive and in-depth understanding of long-term effects. Moreover, diversifying the participants’ age range in future research may enrich the understanding of the impacts throughout development and the generalization of the results to broader age groups.

Another point to consider is the recommendation for replicating this research with a substantially larger number of participants. Expanding the sample would allow for a more comprehensive analysis of the effects of the DHACA method, providing a basis for evidence-based practices. Continuing this research is essential to validate and consolidate its findings, giving greater reliability to the results.
