# Supplementary material for: The effects of the DHACA method on expressive communication in children with autism spectrum disorder
Source: Codas. 2025 Apr 7;37(3):e20240148. doi: 10.1590/2317-1782/e20240148en (PMC11991719; doi:10.1590/2317-1782/e20240148en)
Supplement: Translation

# Translation: Os efeitos do método DHACA na comunicação expressiva em crianças com transtorno do espectro do autismo

**DOI:** 10.1590/2317-1782/e20240148pt

**Authors:** Fernanda Cristina de Oliveira Luna Barbosa, Ana Cristina de Albuquerque Montenegro, Bianca Arruda Manchester de Queiroga

**Affiliations:** 1 Programa de Pós-Graduação em Saúde da Comunicação Humana – PPGSCH, Universidade Federal de Pernambuco – UFPE - Recife (PE), Brasil.

**Keywords:** Autismo, Comunicação, Fonoaudiologia, Tecnologia Assistiva, Sistemas de Comunicação Alternativos e Aumentativos

## Abstract

**Objetivo:**

O objetivo deste estudo foi avaliar as contribuições do método DHACA no desenvolvimento da Comunicação Expressiva em crianças com Transtorno do Espectro do Autismo (TEA).

**Método:**

Trata-se de uma pesquisa longitudinal, do tipo série de casos. A amostra foi constituída por 12 crianças com TEA, com comunicação não verbal ou minimamente verbal, nível de suporte um ou dois. Os dados do estudo foram obtidos por meio da aplicação do Protocolo ACOTEA-R a partir da análise de vídeos gravados das sessões de intervenção antes e após a utilização do Método DHACA. Foram realizadas 20 (vinte) sessões individuais de atendimento fonoaudiológico utilizando o Método DHACA.

**Resultados:**

Após intervenção com o protocolo ACOTEA-R, houve um avanço geral nas habilidades de comunicação expressiva de 10 dos 12 participantes. Quanto ao perfil comunicativo, inicialmente 10 crianças eram não verbais e 02 minimamente verbais. Após a intervenção, 07 evoluíram para o padrão verbal, enquanto 05 crianças permaneceram não verbais. Destaca-se o progresso das seguintes habilidades comunicativas: utilização de frases com quatro ou mais palavras, nomeação de objetos, expressões sociais, cumprimento de pessoas e produção de comentários. Evidenciou-se, ademais, que 08 dos 12 participantes conseguiram avançar até a terceira habilidade do Método DHACA, caracterizada pelo Pedido com Ampliação Lexical e Morfossintática.

**Conclusão:**

Evidenciou-se evolução no desenvolvimento da comunicação expressiva das crianças, tanto por meio do uso do livro de comunicação quanto pela fala, após intervenção com o método DHACA.

## INTRODUÇÃO

O Transtorno do Espectro do Autismo (TEA) é um distúrbio do neurodesenvolvimento que afeta uma a cada 36 crianças no mundo, caracterizado por prejuízo persistente na comunicação social recíproca e na interação social, além de padrões restritos e repetitivos de comportamento, interesses ou atividades^(1)^.

O comprometimento da comunicação no TEA é uma das características que devem estar presentes para ser dado o diagnóstico, podendo ocorrer atraso no desenvolvimento ou mesmo ausência completa da linguagem oral. Estudos destacam, como prejuízos importantes e característicos do TEA, os déficits de comunicação^(2)^. As maiores dificuldades de linguagem enfrentadas por crianças com TEA estão associadas aos aspectos pragmáticos e à estruturação de narrativas de modo que a dificuldade de comunicação social é considerada ponto central dos prejuízos no TEA^(3)^.

Estima-se que aproximadamente 30% das crianças com TEA permaneçam com uma comunicação não verbal ou minimamente verbal, mesmo após anos de intervenção. Embora ainda exista uma limitação na definição precisa do termo “minimamente verbal”, estudos recentes têm conceituado como sendo indivíduos com comunicação expressiva extremamente restrita, e que se comunicam predominantemente por palavras isoladas ou gestos^(4)^.

Nesse cenário, surge a necessidade de disponibilizar a essas crianças um recurso comunicativo alternativo que propicie o início, a manutenção e a expansão das interações dialógicas. Tal recurso deve levar em consideração as inabilidades específicas de atenção compartilhada, direcionamento do olhar e a ausência de intencionalidade presentes no quadro clínico desses indivíduos^(5)^. Essa abordagem se revela fundamental, uma vez que contribui de maneira significativa para preencher lacunas na comunicação, fomentando, assim, uma participação mais ativa e inclusiva em contextos sociais.

A Comunicação Aumentativa e Alternativa (CAA) é uma forma de Tecnologia Assistiva (TA) que proporciona maior autonomia e independência. Trata-se de uma área do conhecimento que oferece uma variedade de técnicas, recursos e estratégias para compensar e facilitar, de maneira temporária ou permanente, as dificuldades de compreensão e expressão comunicativa em indivíduos com graves distúrbios^(6)^.

Para efetivação deste estudo foi utilizado o método DHACA - Desenvolvimento das Habilidades da Comunicação no Autismo, que é um instrumento de CAA criado com o propósito de estimular e desenvolver a comunicação funcional. O Método DHACA privilegia o atendimento fonoaudiológico de crianças com TEA sem fala funcional e tem como pressuposto teórico a Teoria Sociopragmática, a qual enfatiza aspectos sociopragmáticos nos processos de aquisição e desenvolvimento de competências linguísticas^(7-11)^.

O Método DHACA é pioneiro no cenário brasileiro, no que concerne a originalidade no uso de um sistema robusto de comunicação aumentativa e alternativa para desenvolvimento da comunicação funcional de indivíduos com TEA, uma lacuna nas pesquisas nacionais. A implementação desse método visa proporcionar um meio de comunicação mais eficaz, contribuindo significativamente para o processo de aprendizagem, inclusão social e melhoria da qualidade de vida não apenas dos indivíduos com TEA não verbais, mas também de suas famílias.

A flexibilidade inerente ao DHACA, que possibilita adaptações às necessidades individuais de cada criança, é também um diferencial importante, considerando a diversidade de casos encontrados no TEA. Diferentemente de muitos métodos estrangeiros utilizados no país, o DHACA oferece uma opção culturalmente relevante e acessível, alinhando-se à realidade brasileira e ampliando as possibilidades de sucesso nas intervenções^(8-10)^.

O Método DHACA parte do pressuposto de que os sinais linguísticos são adquiridos por meio das interações diárias entre os sujeitos, sendo seu desenvolvimento garantido pela atenção compartilhada e pela compreensão gradual dos interlocutores como agentes intencionais. O DHACA detalha as habilidades comunicativas a serem desenvolvidas com uso de livro com um sistema robusto de comunicação alternativa^(11)^ (Figura 1).

**Figura 1:** Livro DHACA

**Fonte**: A autora (2023)

O presente estudo teve como objetivo avaliar os efeitos do método DHACA no desenvolvimento da comunicação expressiva em crianças com TEA.

## MÉTODO

Trata-se de uma pesquisa longitudinal, do tipo série de casos, de abordagem quantitativa, haja vista que acompanha a evolução dos dados ao longo do período investigado.

Este estudo está integrado ao projeto de pesquisa da Universidade Federal de Pernambuco (UFPE) intitulado “FONOAUDIOLOGIA E AUTISMO - CONHECER, INTERVIR E INCLUIR” registrado e analisado pelo Comitê de Ética da Universidade Federal de Pernambuco, de acordo com a resolução Nº. 466/12 do Conselho Nacional de Saúde, sendo aprovado sob o protocolo Nº 2.106.800.

A presente investigação foi conduzida em uma Clínica-Escola de Fonoaudiologia, no período entre os meses de março a outubro de 2022, onde todos os procedimentos foram integralmente executados. A amostra do estudo foi composta por doze crianças com TEA, com idades entre 3 e 6 anos, das quais 2 eram do sexo feminino (17%) e 10 do sexo masculino (83%).

Foram estabelecidos como critérios de inclusão os seguintes parâmetros: idade compreendida entre 3 e 6 anos, considerando a pertinência de intervir durante a fase de desenvolvimento da linguagem na primeira infância; diagnóstico de TEA, conforme os critérios delineados pela Associação Americana de Psiquiatria no DSM-V (2015)^(12)^.

Como critérios de exclusão, as crianças com TEA com perfil comunicativo caracterizado por uma comunicação verbal ou que apresentavam comorbidades, tais como malformações e/ou síndromes genéticas associadas, bem como alterações neurológicas, deficiências físicas, intelectuais, auditivas ou visuais.

Todos os pais ou responsáveis dos participantes foram devidamente informados sobre os procedimentos metodológicos adotados no estudo, e de forma voluntária, assinaram o Termo de Consentimento Livre e Esclarecido, além de responderem ao protocolo de anamnese e ao protocolo de Avaliação de Itens de Preferência (AIP)^(13)^.

Cada criança foi submetida à Avaliação da Comunicação em Crianças com Transtorno do Espectro do Autismo - ACOTEA-R^(14)^.

O período de intervenção compreendeu um total de vinte sessões de terapia fonoaudiológica individual, realizadas semanalmente, cada uma com a duração de 45 minutos. Cada sessão foi minuciosamente planejada e estruturada pelos terapeutas, levando em consideração as preferências de cada criança, fundamentando-se nas informações colhidas no Protocolo de Avaliação de Itens de Preferência (AIP), administrado antes do início das intervenções, com o propósito de personalizar o processo terapêutico conforme as características específicas de cada participante.

As sessões são realizadas por estudantes de graduação e pós-graduação em fonoaudiologia da UFPE, capacitados para o uso do método DHACA, sob supervisão dos profissionais que idealizaram o referido método (que observavam a intervenção por “espelho espião”) empregando atividades lúdicas semiestruturadas, com o intuito de promover um ambiente propício ao desenvolvimento das habilidades comunicativas, no qual os participantes eram estimulados a se engajar ativamente nas interações comunicativas^(11)^. Foi utilizada a versão do livro de comunicação DHACA, composto por sessenta e seis pictogramas do vocabulário essencial, numa única página, e páginas menores, sobrepostas, com uma linha composta por dez pictogramas de vocabulário acessório, dispostos em páginas separadas de acordo com a categoria lexical, inseridas paulatinamente, durante o processo terapêutico.

Durante as atividades, os itens de interesse dos participantes eram posicionados dentro de seu campo de visão, e eles eram incentivados a se expressar apontando para os pictogramas pertinentes no livro de comunicação. Inicialmente, empregou-se dica física total para ensinar a apontar os pictogramas que representassem a frase inicial, sendo essa gradualmente substituída por dicas física parcial, visuais e verbais, até a criança realizar com independência. Adicionalmente, utilizou-se a estratégia de modelagem, na qual o interlocutor poderia demonstrar a construção da frase desejada, fornecendo o modelo, até que o participante conseguisse se expressar de forma autônoma, sem necessidade de assistência, bem como conversava com a criança utilizando diversas frases com o livro de comunicação DHACA.

O uso de orientações tangíveis, como é o caso da ajuda ou dica física total, dica física leve, dica gestual, otimiza a eficácia no processo de ensino de novas habilidades em crianças diagnosticadas com TEA. Essa abordagem não apenas assegura uma compreensão mais abrangente, mas também fomenta a motivação para a realização das atividades propostas. Tal estratégia leva em consideração a propensão dessas crianças para interagir de forma efetiva com estímulos visuais. As dicas visuais visam à redução de erros, bem como, à ampliação do acesso aos itens de preferência. O processo de esvanecimento das dicas estava condicionado ao desempenho individual de cada participante^(11,15)^.

Vale salientar que antes de iniciar a intervenção, houve um encontro com os pais, com o objetivo de explanar sobre o TEA, sobre Comunicação Aumentativa e Alternativa, tirar dúvidas e por fim, realizar uma oficina prática sobre método DHACA, com uso do livro DHACA entre eles, utilizando a modelagem e as dicas física, visuais e verbais. Além disso, semanalmente, em todas as sessões, os pais conversavam com os terapeutas, momento em que davam o retorno sobre o desenvolvimento do filho em casa e na escola, assim como também eram orientados sobre modelagem, as habilidades que estavam sendo estimuladas e a generalização do uso em diversas situações comunicacionais. Muitas vezes eram propostas atividades junto aos pais, durante a sessão e em seguida, recebiam o feedback.

O Método DHACA apresenta uma descrição detalhada das habilidades comunicativas, conforme descrição a seguir, com seus objetivos específicos. Com a finalidade de adquirir tais habilidades, os participantes eram estimulados a desenvolvê-las, de maneira sequencial^(11)^.

Intenção comunicativa inicial - A criança deve ser capaz de apresentar intenção comunicativa solicitando algo ao interlocutor, apontando para os pictogramas EU + QUERO (no livro de comunicação) + pictograma avulso*.

Pedido com ampliação lexical no vocabulário acessório - A criança deve ser capaz de solicitar algo ao interlocutor, apontando para os pictogramas EU + QUERO + um pictograma do vocabulário opcional de uma das duas abas de categorias lexicais distintas.

Pedido com ampliação lexical e morfossintática - A criança é capaz de formar frases com os pictogramas: EU + QUERO + dois pictogramas (pode ser do vocabulário ativo ou do vocabulário essencial).

Ampliação morfossintática, lexical e das funções comunicativas - Uma criança é capaz de formar frases com três ou mais palavras, com objetivos distintos: Desenvolvimento das funções comunicativas: 1 Função de informação/função interrogativa, com uso de perguntas com os pronomes interrogativos (quem, quando, qual, onde, etc.); 2 Função de comentários: realizar comentários, informações espontâneas, demonstrar algo, demonstrar dor, dar opinião, expressar ideias; 3. Função de expressar sentimentos, gratidão; 4. Função sociointerativa: realizar saudações, despedidas, agradecimentos, pedir desculpas e se exibir. A construção da frase ocorre de forma sequenciada, apontando para os pictogramas, podendo ser acompanhada da fala. 5. Diálogo - A criança é capaz de apresentar as seguintes funções comunicativas: Função de relato: contar um fato ou recontar estórias; Função imaginativa: criar uma história ou contar piadas; Função conversacional: sustentar uma conversa.

Após o período de intervenção, os participantes da pesquisa foram submetidos a uma reavaliação. Os dados foram coletados utilizando o protocolo ACOTEA-R^(14)^.

Para a coleta de dados procedeu-se à gravação das três primeiras sessões, bem como das três últimas sessões de cada participante da pesquisa. Os vídeos resultantes tiveram uma média de duração de 45 minutos cada. Essa análise teve por objetivo examinar os comportamentos comunicativos, por meio da aplicação do protocolo ACOTEA-R^(14)^.

A sistematização dos dados encontrados foi conduzida pela pesquisadora responsável, englobando a avaliação dos registros contidos nas anamneses, nos protocolos e nas fichas de evoluções geradas ao longo das sessões de fonoaudiologia, além da análise das gravações em vídeo das avaliações realizadas no ambiente terapêutico para preenchimento do protocolo ACOTEA-R^(14)^. Os dados obtidos foram devidamente registrados e sistematizados em uma planilha digital utilizando o software Microsoft Excel. Na etapa subsequente, realizou-se uma análise estatística descritiva de frequência de respostas para melhor interpretação dos resultados. Cada habilidade comunicativa a ser avaliada no protocolo ACOTEA-R^(14)^ foi codificada com a letra P seguida do número (Ex: P1, P2, P3).

## RESULTADOS

Será apresentada aqui a análise dos dados coletados referentes aos escores das habilidades comunicativas expressivas que constam no Protocolo ACOTEA-R^(14)^ aplicado antes e após a intervenção com o uso do método DHACA.

Na Figura 2, observa-se o desenvolvimento da produção de fala. Na Figura 3, como é possível observar, houve um aumento na pontuação obtida no Protocolo ACOTEA-R^(14)^ em 10 dos 12 participantes. Isso evidencia que a intervenção utilizando o Método DHACA promoveu o desenvolvimento das habilidades comunicativas expressivas na grande maioria dos participantes.

**Figura 2:** Resultado do perfil comunicativo pré e pós-intervenção com Método DHACA. Recife, 2024

Não verbal (NV); Minimamente verbal (MV); Verbal (V); Sexo – Masculino (1); Feminino (2)

**Figura 3:** Escores das Habilidades comunicativas expressivas das crianças com TEA antes e após intervenção com o Método DHACA

**Fonte**: A autora (2023)

Legenda das habilidades de comunicação expressiva avaliadas no ACOTEA-R: P1 = Expressa incomodo; P2 = Protesta para mostrar que não quer algo; P3 = Demonstra que gosta das coisas; P4 = Expressa interesse em outras pessoas; P5 = Solicita para que seja dado continuidade quando uma atividade, gesto ou brincadeira é interrompida; P6 = Solicita quando é retirado algum objeto dela ou quando ela quer mais algum alimento; P7 = Chama a sua atenção; P8 = Solicita algo que que você nunca lhe tenha oferecido, mas que esteja dentro do seu alcance visual, auditivo, tátil; P9 = Solicita objetos que não estejam à vista; P10 = Cumprimenta as pessoas; P11 = Oferece coisas ou as compartilha; P12 = Mostra algo a você; P13 = Pede ajuda; P14 = Utiliza produções sociais (oi, obrigada, tchau); P15 = Faz perguntas; P16 = Nomeia objetos ou pessoas, espontaneamente ou em resposta a sua pergunta; P17 = Faz comentários; P18 = Usa frases com quatro ou mais palavras; P19 = Respeita turnos e mantém uma conversa; P20 = NÃO Apresenta birras; P21 = Sorrir social

Apesar de dois participantes não apresentarem progressos significativos nas pontuações do Protocolo ACOTEA-R^(14)^, eles manifestaram melhoria em habilidades específicas. Um dos participantes apresentou avanços em cinco habilidades anteriormente manifestadas por meio de estereotipias motoras e verbais, que não foram mais evidenciados nessas mesmas situações. Além disso, houve uma redução na prática de empurrar objetos para solicitar algo por parte do outro participante. Embora não tenha havido uma melhoria significativa nas habilidades comunicativas expressivas do Protocolo ACOTEA-R^(14)^ para esses participantes, houve ganho na comunicação expressiva para ambos.

A análise dos dados referentes a cada habilidade de comunicação expressiva do Protocolo ACOTEA-R^(14)^ (Figura 4), ressalta o progresso observado nas seguintes habilidades comunicativas, por meio do aumento da frequência:

**Figura 4:** Escores Total das Habilidades comunicativas expressivas antes e após intervenção com o Método DHACA

**Fonte**: A autora (2023)

P18 - Utilizar frases com quatro ou mais palavras: de 0 para 17;
P16 - Nomear objetos ou pessoas: de 2 para 18;
P10 - Cumprimentar as pessoas: de 1 para 14;
P17 - Fazer comentários: de 0 para 7.

Na Figura 3, evidencia-se uma evolução nas formas de resposta observadas. Notavelmente, a categoria “Fala” surgiu como um indicador marcante de progresso, transpondo uma frequência inicial de 3 atingindo a frequência final de 28, merece destaque, da mesma forma, as demais respostas como “Apontar figura com uso do livro sem fala” (frequência de 0 para 33); “Apontar figura com uso do livro com fala” (frequência de 3 para 28); “Gestos” (frequência de 16 para 36) e “Entrega algo” que apresentou um aumento de frequência de 20 para 30 (Figura 3).

Por outro lado, é importante mencionar a relevante redução nos índices das respostas consideradas menos funcionais (Figura 5). Observou-se uma diminuição nas frequências de comportamentos como “Estereotipias motoras”, que sua frequência declinou de 37 para 20; “Estereotipias verbais” (frequência de 22 para 15); “Puxa o outro” (frequência de 26 para 21) e “Grito/birra” manifestaram uma redução saindo de frequências iniciais de 20 para 15, destacando a eficácia da intervenção na minimização de comportamentos menos adaptativos.

**Figura 5:** Modos de expressão para as habilidades comunicativas expressivas do ACOTEA-R antes e após intervenção. Recife, 2024

**Fonte**: A autora (2023)

Dentre os aspectos analisados após a intervenção fonoaudiológica, destacam-se progressos no desenvolvimento das habilidades na comunicação expressiva, evidenciados por um aumento e surgimento de fala, comunicação por meio da CAA apontando figuras (pictogramas) com uso do livro de comunicação DHACA com e sem fala, uso de gestos, bem como a entrega de objetos para expressar o desejo de alcançar ou obter algo, além do contato visual, conforme demonstrado na Figura 5.

De acordo com a Figura 5, é relevante destacar o progresso na produção da fala funcional, seja de maneira espontânea ou por intermédio da CAA, utilizando figuras (pictogramas) por meio do livro de comunicação DHACA.

No que concerne ao perfil comunicativo, conforme descrito na Figura 2, a seguir, os resultados obtidos por meio da aplicação do Protocolo ACOTEA-R^(14)^ evidenciaram considerável progresso. Inicialmente, apenas duas crianças (16,6%) exibiam um padrão de comunicação minimamente verbal, enquanto dez crianças (83,3%) apresentavam um perfil não verbal, de acordo com a avaliação inicial. Entretanto, os dados resultantes da reavaliação do Protocolo ACOTEA-R^(14)^ após as vinte sessões de intervenção revelaram que cinco crianças (41,6%) mantiveram um perfil comunicativo não verbal, enquanto sete crianças (58,3%) fizeram a transição para um perfil comunicativo verbal.

No contexto do desenvolvimento das habilidades comunicativas estabelecidas pelo Método DHACA, houve um progresso significativo. Após a intervenção, notavelmente, 66,67% dos participantes conseguiram avançar até a terceira habilidade do Método, caracterizada pelo Pedido com Ampliação Lexical e Morfossintática, enquanto dois (16,67%) participantes alcançaram a penúltima habilidade, que envolve a Ampliação Morfossintática, Lexical e das Funções comunicativas, além de que 16,67% das crianças desenvolveram até a segunda habilidade “Pedido com Ampliação Lexical” no vocabulário acessório.

## DISCUSSÃO

Os resultados do presente estudo revelaram avanços na comunicação expressiva em crianças com o transtorno do espectro autismo após a intervenção pelo Método DHACA^(11)^. Essa evolução foi delineada não apenas através do aumento na pontuação do escore geral do protocolo ACOTEA-R^(14)^, mas também na identificação das habilidades comunicativas expressivas que se revelaram com maior frequência entre os participantes do estudo. Essas descobertas proporcionam uma visão do progresso alcançado por essas crianças, destacando os impactos positivos e os avanços observados em sua capacidade de comunicação expressiva.

Houve um aumento nesses escores em 83,33% dos participantes. Isso evidencia que a intervenção com o Método DHACA^(11)^ promoveu, com o uso de ferramentas da CAA, o desenvolvimento das habilidades da comunicação expressiva nesta população do estudo, corroborando com a literatura internacional^(16)^.

De modo semelhante, um estudo de caso recente investigou o potencial da tecnologia assistiva para colaborar na melhoria das habilidades de comunicação em indivíduos que enfrentam tais dificuldades, resultando em um progresso significativo nas áreas comunicativas do participante. O estudo também destacou um avanço marcante nas interações sociais, evidenciando o emprego de formas cordiais de comunicação. Além disso, foi observada uma redução na gravidade dos sintomas do TEA apresentados pela criança, que passou de sintomas moderados a leves dentro do espectro autista^(17)^.

As habilidades de comunicação e de interação social são descritas como as mais prejudicadas em crianças com TEA^(18)^ No caso do presente estudo, os dados apresentados na Figura 4 revelam uma evolução nas funções comunicativas, resultando em uma comunicação mais funcional, apresentando as funções: fazer comentários, mostrar algo ao outro, cumprimentar pessoas, utilizar produções sociais (p. ex.: oi, tchau, ...).

Quanto aos aspectos gramaticais, duas crianças, atingiram a segunda habilidade do Método DHACA, “Pedido com Ampliação Lexical no Vocabulário Acessório”, (Eu+Quero + pictograma); 8 crianças alcançam “Pedido com Ampliação Lexical e Morfossintática” (eu + quero + duas palavras) e duas crianças atingiram a quarta habilidade: “Ampliação morfossintática, lexical e das funções comunicativa”, com frases com quatro ou mais palavras e diversas funções comunicativas. Na Figura 4 foi observado um aumento significativo sobretudo na habilidade P18 do ACOTEA (“Uso de frase com quatro ou mais palavras”) em 50% das crianças, confirmando um grande desenvolvimento, já que antes da intervenção, não havia registro de nenhuma criança demonstrando essa habilidade.

É importante destacar que, em uma pesquisa cujo escopo foi a investigação de atrasos na sintaxe, morfologia e vocabulário no TEA, constatou-se que as crianças apresentavam prejuízos nas habilidades sintáticas e morfológicas^(19)^. Assim, a habilidade de utilizar, de maneira funcional, frases contendo quatro ou mais palavras – competência delineada no desenvolvimento do Método DHACA – emerge como indicativo de que tal método promove efetivamente o desenvolvimento da competência gramatical.

Neste sentido, afirma-se que, o aparecimento de frases mais extensas, pode ser compreendido como a aquisição de uma sintaxe mais complexa. É importante destacar que essa competência linguística está relacionada à comunicação social, afetando a interação social recíproca^(20)^.

Esses resultados indicam um avanço na comunicação expressiva evidenciando uma maior robustez linguística após a implementação do Método DHACA. Este fenômeno corrobora as conclusões de uma revisão que sustenta que a intervenção com CAA propicia, como resultado positivo, o desenvolvimento comunicativo^(16)^

Ademais, destaca-se que tal intervenção não apenas incide sobre a comunicação não verbal, mas também impulsiona o desenvolvimento da produção oral de palavras e frases. E, no presente estudo, o desenvolvimento da fala funcional aparece em 5 crianças com ou sem o apoio do livro de comunicação DHACA.

Conforme demonstrado, as habilidades comunicativas do Método DHACA^(11)^, que apresentaram níveis mais elevados de aquisição e/ou progresso após a intervenção, incluem: a capacidade de solicitar a continuidade de atividades ou brincadeiras, requisitar objetos quando removidos ou manifestar o desejo por mais alimentos, solicitação de ajuda, cumprimentar pessoas, empregar produções sociais, nomear objetos de maneira espontânea ou em resposta a questionamentos e utilizar frases compostas por quatro ou mais palavras.

Importante ressaltar que as habilidades de “solicitar” (solicitar a continuidade de atividades ou brincadeiras, requisitar objetos quando removidos ou manifestar o desejo por mais alimentos, solicitação de ajuda) embora seja uma das primeiras funções pragmáticas, surge com frequência significativa com uso da fala ou do CAA ou de modo misto, demonstrando a evolução da linguagem enquanto uso de um sistema linguístico.

No que concerne à competência de nomear objetos, pessoas ou atributos espontaneamente, observou-se um aumento na frequência dessas ocorrências. Os dados demonstraram um crescimento significativo, passando de apenas uma criança realizando a nomeação de objetos antes da intervenção para sete crianças que adquiriram tal habilidade após a implementação do Método DHACA^(11)^.

A habilidade de nomear objetos infere que há uma compreensão acerca do comando do outro, revelando a capacidade da criança em responder adequadamente à solicitação para nomear algo. Este processo evidencia não apenas a habilidade de nomear, mas também demonstra uma compreensão da intenção comunicativa do outro dentro de um contexto. Acresce a isso, presença significativa das habilidades e de cumprimentar pessoas e utilizar expressões sociais, que refletem uma interação social dinâmica, em que tanto a criança quanto o adulto/terapeuta compreendem a intenção do comportamento (ação intencional) e do estado intencional um do outro (interesses comunicativos).

Sabe-se que o uso de CAA é implementado a partir da interação com o outro^(11)^. As crianças desta pesquisa não utilizavam nem CAA nem a fala para se comunicar. Observa-se que houve o desenvolvimento da comunicação expressiva após intervenção com o método DHACA^(11)^, surgindo a comunicação mista, caracterizada pela utilização simultânea de categorias comunicativas (produção de frases por meio da combinação do livro de comunicação com fala de maneira simultânea)^(21)^.

De acordo com a Figura 2, referente às habilidades de comunicação expressiva, os resultados constatam o desenvolvimento da produção de fala, os quais corroboram os dados evidenciados em uma pesquisa meta-analítica, cujo escopo consistiu em avaliar o impacto da Comunicação Aumentativa e Alternativa (CAA) na produção da fala de indivíduos com deficiência de desenvolvimento. Este levantamento englobou 23 estudos, envolvendo 67 sujeitos, e verificou de modo consistente que a grande maioria (89%) experimentou avanços significativos na produção de fala após a implementação da CAA^(22)^.

Nota-se uma promoção no desenvolvimento da fala funcional, com um acréscimo de 28 nas frequências de respostas após a intervenção. Anteriormente à intervenção, essa habilidade não era demonstrada, corroborando com as descobertas dos autores^(22)^, que em suas análises, apresentaram dados sugerindo que as intervenções que utilizam a CAA também podem ter efeitos positivos na produção natural da fala.

A análise abrangente dos dados de desenvolvimento da comunicação funcional (Figura 5) destaca a redução de comportamentos comunicativos mais primários, como gritos, choros e birras. Este achado corrobora com a pesquisa conduzida recentemente que teve como propósito investigar a perspectiva de professores de educação especial em relação às barreiras e facilitadores no uso da Comunicação Alternativa e Aumentativa com alunos portadores de deficiência múltipla^(22)^. O autor ressalta a presença de comportamentos desafiadores frequentemente associados a dificuldades de comunicação. Comumente, indivíduos que enfrentam obstáculos na expressão de suas necessidades recorrem a esses comportamentos como forma de comunicação, o que por sua vez dificulta as relações sociais.

Esse estudo corrobora com estudo^(23)^ que demonstra a eficácia da comunicação alternativa enquanto tecnologia assistiva com potencial para estimular o desenvolvimento da fala, além de facilitar a aquisição de competências linguísticas e contribuir para o processo de aprendizagem. Ademais, o estudo realça os impactos significativos da CAA na promoção da inclusão social.

Estudos prévios, que têm destacado o uso de ferramentas de comunicação alternativa para melhorar as habilidades comunicativas de crianças com TEA, além de estimular a comunicação espontânea^(24)^ e, os dados desta pesquisa fortalecem a afirmação de que demonstram os avanços na produção da fala funcional, tanto de forma autônoma, quanto com o suporte do livro de comunicação DHACA, como mostrado na Figura 5.

Com base no exposto, pode-se inferir que o método DHACA^(11)^, apresenta-se como uma abordagem terapêutica, nacional inovadora para desenvolver a comunicação expressiva, e de modo especial, a morfossintaxe, de crianças com TEA.

Ademais, um estudo de revisão foi conduzido com o objetivo de sintetizar as evidências contemporâneas sobre a eficácia da Comunicação Aumentativa e Alternativa (CAA), os resultados destacam melhorias importantes na fala e na esfera pragmática quando se utiliza o sistema de comunicação alternativo. Observa-se também que as crianças que receberam intervenção por meio da CAA apresentaram ganhos significativamente maiores em vocabulário funcional, em comparação com seus pares que não foram submetidos a esse método^(25)^.

De acordo com o Método DHACA^(11)^, a continuidade das atividades em casa, o engajamento da família às orientações estabelecidas, a utilização do livro DHACA na interação dialógica entre a criança e os parceiros de comunicação no dia a dia são aspectos essenciais que influenciam os resultados obtidos. Neste sentido, foi observado que dois participantes não apresentaram evolução significativa. A ausência de continuidade do uso do livro DHACA em casa, relatada pelos pais e cuidadores dessas crianças (registradas semanalmente nas fichas de evolução, logo após a sessão), pode ter impactado negativamente o progresso dessas crianças, uma vez que o envolvimento regular e consistente é crucial para a consolidação das habilidades adquiridas durante as sessões de intervenção. A falta de evolução significativa nessas duas crianças destaca a necessidade de uma abordagem colaborativa na implementação de programas de intervenção em CAA, enfatizando a importância da participação ativa da família e da generalização das habilidades aprendidas no contexto do dia a dia da criança.

Essa constatação reflete os achados de outro estudo recente, o que enfatizou que o sucesso das intervenções em crianças com TEA está intrinsecamente ligado ao apoio e à participação ativa da família no processo. O envolvimento dos pais não apenas facilita a aprendizagem e a aplicação de um sistema de Comunicação Alternativa, mas também promove uma maior generalização das habilidades de comunicação para diferentes contextos e situações do dia a dia^(26)^.

O tamanho da amostra pode implicar em uma representação limitada da diversidade de respostas, restringindo a extensão da generalização dos achados. Portanto, para uma compreensão mais abrangente e conclusiva dos impactos do Método DHACA na comunicação expressiva de crianças com TEA, recomenda-se novos estudos com uma amostra mais ampla e heterogênea.

## CONCLUSÃO

Os resultados desta pesquisa revelam o papel significativo desempenhado pela implementação do Método DHACA no avanço das habilidades de comunicação expressiva em crianças com Transtorno do Espectro Autista, corroborando a hipótese inicial que norteou este estudo. Os resultados apontam efeito positivo do Método DHACA no desenvolvimento da comunicação expressiva em crianças com TEA, demonstrados na aquisição de fala, no desenvolvimento morfossintático e da intenção comunicativa, bem como em outras funções pragmáticas. Esses resultados não apenas confirmam a eficácia do Método DHACA, mas também indicam seu impacto positivo.

Contudo, é preciso reconhecer as limitações inerentes a esta pesquisa, os achados apresentados neste estudo não representam uma conclusão definitiva, mas um ponto de partida para futuras investigações mais aprofundadas. A duração da intervenção, embora tenha proporcionado resultados positivos, sugere a necessidade de investigações mais prolongadas para uma compreensão mais abrangente e aprofundada dos efeitos a longo prazo. Além disso, a diversificação da faixa etária dos participantes em futuras pesquisas pode enriquecer a compreensão dos impactos ao longo do desenvolvimento e da generalização dos resultados para grupos etários mais amplos.

Outro ponto de consideração é a recomendação para a replicação desta pesquisa com um número substancialmente maior de participantes. A expansão do universo amostral permitirá uma análise mais abrangente dos efeitos do Método DHACA, proporcionando uma base para as práticas baseadas em evidências. A continuidade dessa investigação é essencial para validar e consolidar as descobertas apresentadas, conferindo maior confiabilidade aos resultados.
